# Whole transcriptome analysis of adrenal glands from prenatal glucocorticoid programmed hypertensive rodents

**DOI:** 10.1038/s41598-020-75652-y

**Published:** 2020-10-30

**Authors:** Sujeenthar Tharmalingam, Sandhya Khurana, Alyssa Murray, Jeremy Lamothe, T. C. Tai

**Affiliations:** 1 Northern Ontario School of Medicine, Laurentian University, 935 Ramsey Lake Rd, Sudbury, ON P3E 2C6 Canada; 2 Department of Biology, Laurentian University, Sudbury, ON P3E 2C6 Canada; 3 Department of Chemistry and Biochemistry, Laurentian University, Sudbury, ON P3E 2C6 Canada; 4 Biomolecular Sciences Program, Laurentian University, Sudbury, ON P3E 2C6 Canada; 5 Health Sciences North Research Institute, Sudbury, ON P3E 2H2 Canada

**Keywords:** Cell biology, Circadian rhythms, Molecular biology, Transcriptomics, Adrenal cortex hormones, Physiology, Cardiovascular diseases, Hypertension

## Abstract

Prenatal glucocorticoid exposure is associated with the development of hypertension in adults. We have previously demonstrated that antenatal dexamethosone (DEX) administration in Wistar-Kyoto dams results in offspring with increased blood pressure coupled with elevated plasma epinephrine levels. In order to elucidate the molecular mechanisms responsible for prenatal DEX-mediated programming of hypertension, a whole-transcriptome analysis was performed on DEX programmed WKY male adrenal glands using the Rat Gene 2.0 microarray. Differential gene expression (DEG) analysis of DEX-exposed offspring compared with saline-treated controls revealed 142 significant DEGs (109 upregulated and 33 downregulated genes). DEG pathway enrichment analysis demonstrated that genes involved in circadian rhythm signaling were most robustly dysregulated. RT-qPCR analysis confirmed the increased expression of circadian genes *Bmal1* and *Npas2*, while *Per2*, *Per3*, *Cry2* and *Bhlhe41* were significantly downregulated. In contrast, gene expression profiling of Spontaneously Hypertensive (SHR) rats, a genetic model of hypertension, demonstrated decreased expression of *Bmal1* and *Npas2*, while *Per1*, *Per2*, *Per3*, *Cry1*, *Cry2*, *Bhlhe41* and *Csnk1D* were all upregulated compared to naïve WKY controls. Taken together, this study establishes that glucocorticoid programmed adrenals have impaired circadian signaling and that changes in adrenal circadian rhythm may be an underlying molecular mechanism responsible for the development of hypertension.

## Introduction

Fetal exposure to an unfavourable *in-utero* environment has been strongly associated with the development of numerous adulthood diseases, a phenomenon referred to as fetal programming^1^. In-utero insults such as maternal undernutrition, placental dysfunction, hypoxia and fetal exposure to alcohol, nicotine and glucocorticoids (GCs) are established determinants which contribute to the programming of adult diseases in various species^2–7^. Emerging evidence suggests that these stressors trigger molecular reconfiguration at the cellular level as a compensatory mechanism to survive the *in-utero* insult^4,7^. This adaptation results in permanent molecular changes which increases the risk of developing disease later in life^8,9^. Diseases linked to fetal programming include cardiovascular disease (CVD), kidney disease, adrenal dysfunction, metabolic syndrome, insulin resistance and hypertension^3,7,10–13^.

We have previously shown that antenatal administration of synthetic GCs such as dexamethasone (denoted as DEX) in Wistar-Kyoto (WKY) dams results in offspring that developed increased elevated systolic, diastolic, and mean arterial pressure, along with increased plasma epinephrine levels as adults^7,14^. GCs are lipophilic hormones that can readily cross the placenta resulting in a stress-like *in-utero* environment^15^. The placenta expresses the 11β-dehydroxysteroid dehydrogenase 2 (11β-HSD2) enzyme which metabolizes GCs, therefore normal *in-utero* GC concentrations are substantially reduced in comparison to maternal levels^16^. A recent study demonstrated that placental expression of 11β-HSD2 is rhythmically expressed and that it is possible for rhythmic GC passage through the placental barrier^17^. In addition, elevated fetal GC exposure is observed in pregnancies complicated with pre-eclampsia^18^ or intrauterine growth retardation^19^ where there is reduced placental 11β-HSD2 expression. Furthermore, 11β-HSD2 is ineffective in metabolizing synthetic GCs, therefore DEX is able to pass through the transplacental passage and into the *in-utero* environment^20^. Clinically, synthetic GCs have proven to accelerate fetal lung maturation and is therefore given to pregnant women at risk of preterm birth^21^.

Despite evidence for GC mediated fetal programming of adult hypertension, the underlying molecular mechanisms have been largely uncharacterized. We and others have shown permanent molecular programming of genes involved in the catecholamine biosynthesis pathway in the adult adrenal glands of prenatal DEX exposed WKY rats^4,7,14,22,23^. Here, the programmed adrenal glands demonstrated modest upregulation of *tyrosine hydroxylase*, *dopamine β-hydroxylase* and *phenylethanolamine N-methyl transferase*. Indeed, the adrenal gland is part of the hypothalamic–pituitary–adrenal (HPA) axis which has been implicated with cardiovascular disorders and the development of hypertension^24,25^. The HPA axis contributes to the physiological response to stress, and acts on the adrenal medulla to promote the biosynthesis and secretion of the catecholamine epinephrine, which binds to adrenergic receptors throughout the body resulting in increased blood pressure. To date, a comprehensive global-scale molecular analysis of the DEX programmed adrenal gland has not been established. Identification of global gene expression alterations in the programmed adrenal glands will help elucidate the molecular mechanisms which contribute to the development of hypertension in adulthood.

In this study, a whole-transcriptome analysis was performed on DEX programmed WKY male adrenal glands using the Rat Gene 2.0 microarray (Thermo Fisher Scientific). 142 annotated significantly differentially expressed genes (DEGs) were identified in DEX exposed offspring compared with saline-treated controls (109 upregulated genes and 33 downregulated genes). Pathway enrichment and upstream regulator analyses of the DEG list demonstrated that genes involved in circadian rhythm signaling were most robustly dysregulated. RT-qPCR analysis confirmed the increased expression of circadian genes *Bmal1* and *Npas2*, while *Per2*, *Per3*, *Cry2* and *Bhlhe41* were significantly downregulated in adrenals from DEX exposed animals compared to saline controls. We also determined the gene expression profile of the Spontaneously Hypertensive (SHR) rats^26^. Here, the SHR animals also demonstrated dysregulation of the circadian rhythm but with opposing results to the fetal programming model. Taken together, the overall data suggests that dysregulation of circadian rhythm signaling may be an underlying mechanism for the development of hypertension.

## Methods

### Animals, DEX injections, and tissue collection

WKY (Wistar Kyoto) and SHR (Spontaneously Hypertensive) rats were purchased from Charles River Laboratory (Montreal, QC, Canada) and housed in Laurentian University’s animal care facility. All protocols were approved by the Laurentian University Animal Care Committee in accordance with guidelines from the Canadian Council on Animal Care. Rats were exposed to a 12-h light–dark cycle, with the light phase set between 6:00 am to 6:00 pm. Food and water were available ad libitum.

WKY rats were fetal programmed with DEX as previously shown^4,7^. Briefly, WKY male and female rats (aged 8 weeks) were acclimatized for 2 weeks. One male rat was placed with three female rats. The females were monitored for vaginal plugs daily and housed individually once the plugs were observed. Pregnant females were administered subcutaneous injections of DEX throughout the third trimester (days 15 – 21) at 100 μg/kg/day prepared in 0.9% NaCl with 4% ethanol, or the control saline solution. The naïve rats did not receive injections. The resulting pups were weaned at 3 weeks of age, and 2–3 rats were housed per cage according to sex. In a separate cohort, 17 week old male SHR and WKY rats were purchased and acclimatized for 2 weeks without breeding or injections. At 19 weeks, male rats were anaesthetized by an intraperitoneal administration of 75 mg of ketamine (CDMV Inc) and 5 mg xylazine (Sigma) per Kg of body weight. Adrenal glands were isolated, frozen on dry ice and stored at − 80 °C until further processing. All anesthetizations and adrenal sample collection was performed between 10 to 11 am.

### RNA extraction and cDNA synthesis

Total RNA was extracted from the adrenal glands using TRI Reagent (Sigma) according to manufacturer’s instructions. Briefly, the left adrenal gland was placed in a microfuge tube with 1 mL TRI reagent and one stainless steel bead, and homogenized using a Tissuelyser (Qiagen) for 2 cycles at 30 Hz for 2 min. The homogenized tissue was centrifuged at 12,000×*g* for 10 min at 4 °C. The supernatant was mixed with 200 µl of chloroform (Sigma) and centrifuged. The aqueous phase which contains the RNA was carefully transferred to a fresh microfuge tube, mixed well with 500 µl of isopropanol (Sigma), and centrifuged at 12,000 × g for 8 min at 4 °C. The supernatant was discarded and the pellet was resuspended in 1 ml of 70% ethanol. The tubes were then centrifuged at 7500×*g* for 5 min and the ethanol was discarded. The pellet containing the purified RNA was subsequently air dried and dissolved in diethylpyrocarbonate (DEPC)-treated water. The RNA samples were analyzed using NanoDrop (ND-1000 spectrophotometer) to measure absorbance ratio at 260/280 nm and 260/230 nm in order to assess RNA purity. RNA samples below absorbance ratio of 1.8 were excluded from analysis.

Genomic DNA was removed from the purified RNA samples using the DNAseI kit (Sigma) according to manufacturer’s instructions. The RNA samples were then reverse transcribed using random hexamers (Sigma), mixed dNTPs (VWR), and M-MLV reverse transcriptase (Promega) according to manufacturer’s instructions.

### Primer design and reverse transcribed-quantitative polymerase chain reaction (RT-qPCR)

Forward and reverse primer pair sequences for genes of interest were selected from Primer3 (NCBI). Design criteria for primer sequences included target sequence length between 75–150 base pairs, 50–60% GC content and melting temperatures between 57–63 °C. In addition, primer pairs were selected to span exon–exon junctions to avoid detection of genomic DNA. Primers were custom ordered from Sigma and were validated by plotting critical threshold (C_q_) values against a sevenfold cDNA serial dilution on a logarithmic scale. The reaction efficiency of each primer pair was calculated according to the formula E = [10^(−1/slope)^− 1]. Primers with reaction efficiency between 90 to 110%, and R^2^ value greater than 0.99 were considered validated and acceptable for analysis. In addition, optimal annealing temperature for each primer pair was identified by performing temperature gradient analysis and identifying annealing temperature which resulted in smallest C_q_ value. The complete list of validated primer sequences can be found in Supplementary Table #1.

RT-qPCR reactions were performed using the Quantstudio 5 qPCR instrument (ThermoFisher Scientific) in 15 μL reaction volumes as described previously^27^. All samples were analyzed in duplicate and normalized to three independent control housekeeping genes (*Gapdh*, *Rpl-13* and *Rpl-32*). The relative mRNA transcript level of each gene was reported according to the ΔΔ*C*_*q*_ method as mRNA fold increase: 2^ΔΔ^*C*_*q*_ = 2^(Δ*Ct* gene of interest – Δ*Cq* housekeeping genes)^. For each gene, average 2^ΔΔ^*C*_*q*_ and standard error of means (SEM) for all samples were calculated.

### Whole transcriptome microarray

Total RNA from 18 male adrenal samples (6 naïve, 6 saline and 6 DEX treated rats) were sent to The Centre for Applied Genomics (TCAG) Microarray Facility (The Hospital for Sick Children, ON, Canada) for whole transcriptome profiling. RNA quality was verified on the Agilent 2100 Bioanalyzer (Agilent Technologies) to ensure samples had an RNA integrity number ≥ 8.0 and A:260/280 > 1.95. RNA samples were assayed for whole transcriptome analysis at TCAG utilizing the Rat Gene 2.0 ST GeneChip (ThermoFisher Scientific). The raw microarray data was quality checked, normalized and analyzed utilizing the Transcriptome Analysis Console (TAC) Software 4.0.2.15 (Thermo Fisher) with the rat reference genome (Rnor_5.0) to generate the differential gene expression (DEG) list. DEG selection criteria was set to fold-change < − 1.5 and > 1.5, p-value < 0.05 and false discovery rate < 0.1. TAC was also used to perform exploratory grouping analysis (EGA). The following EGA parameters were utilized: variance filter of 20,000 maximally variant genes non-weighted, t-SNE dimension reduction with perplexity = 4 and affinity clustering with affinity = 0.25.

### Gene ontology (GO) enrichment and pathway analysis

GO enrichment and pathway analysis was performed using iPathwayGuide (iPG; Advaita Bioinformatics). The DEGs were analyzed in the context of pathways obtained from the Kyoto Encyclopedia of Genes and Genomes (KEGG) database (Release 90.0 + /05–29) and the GO Consortium database (2019-Apr26). The “Impact Analysis” approach was utilized to score the GO pathways and FDR correction was applied with p-value threshold set to < 0.05 as previously described^28,29^.

### Upstream regulator analysis

iPG was used to identify the predicted upstream gene regulators. This analysis utilized the experimental DEG enrichment data in combination with iPG’s proprietary knowledge base on regulatory interaction networks. This information was used to compute the Z-score and the corresponding p-value for each upstream regulator as previously demonstrated^30^. Here, the predicted activation or inhibition state of the upstream regulator was provided. Upstream regulators were considered statistically significant when FDR p-value was < 0.05.

### Statistics

The data for the RT-qPCR experiments is presented as mean ± SEM. Between group comparisons were performed using one-way ANOVA followed by Tukey’s post-hoc analysis. Statistical significance was identified for comparisons with p-value < 0.05. Statistical analysis for the microarray data is detailed in the TAC User Guide (assets.thermofisher.com/TFS-Assets/LSG/manuals/tac_user_manual.pdf). The statistical parameters for iPG analyses is outlined here (https://www.advaitabio.com/ipathwayguide).

## Results

### Whole transcriptome analysis

**Figure 1:**
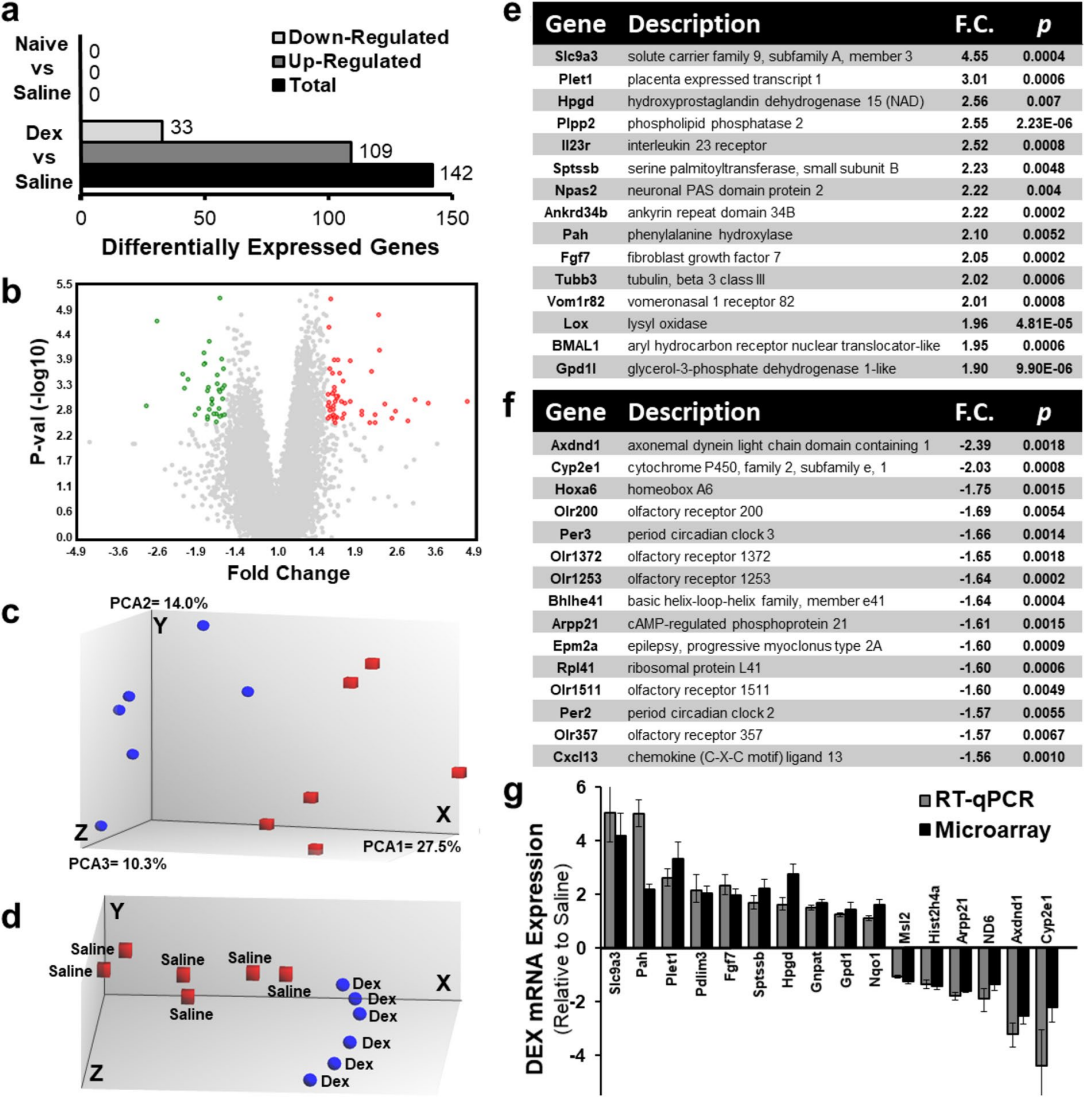
Overview of whole transcriptome microarray analysis in 19-week-old male WKY adrenals exposed to prenatal DEX relative to saline controls. (**a**) The Rat Gene 2.0 ST GeneChip microarray (ThermoFisher Scientific) identified 142 annotated differentially expressed genes (DEGs) in prenatal DEX versus saline-treated samples, with 109 upregulated genes and 33 downregulated genes (criteria: fold-change < − 1.5 and > 1.5; p-value < 0.05; false discovery rate < 0.1). There were no DEGs between naive and saline controls. (**b**) A volcano plot depicting the whole transcriptomic analysis illustrates highly dysregulated genes to the left and right sides of the plot, while genes higher on the graph indicates increased statistical significance. DEGs in DEX versus saline with p-value below 0.05 are marked in red (> 1.5 fold change) and green (< − 1.5 fold change). (**c**) Principal component analysis (PCA) of microarray data. PCA was performed on DEX (blue spheres) and saline (red cubes) datasets and the resulting scores for the first three principal components are presented. The three principal components accounted for 51.8% of the variance in the datasets. This analysis revealed that the DEX and saline samples form distinct groupings. (**d**) Exploratory grouping analysis (EGA) of whole-transcriptome datasets from DEX and saline samples. EGA was performed without pre-defining known sample attributes. Associating the sample IDs to the EGA plot demonstrates clear non-homogeneous distribution of the datasets into two distinct clusters: hypertensive DEX (blue spheres) and normotensive saline controls (red cubes). (**e**) Top 15 upregulated genes and (**f**) top 15 downregulated genes in DEX versus saline datasets presented with p-value (*p*) and ranked based on fold change (F.C.). (**g**) Validation of whole-transcriptomic microarray data via RT-qPCR analysis using 16 representative genes. Genes were randomly selected to include top up and down-regulated DEGs as well as moderate DEGs. Relative gene expression depicted as fold change is shown for both the microarray and RT-qPCR assays. Comparison of fold changes between RT-qPCR and microarray were generally similar and in the same order of magnitude.

In order to elucidate the molecular mechanisms responsible for prenatal DEX-mediated hypertension, whole-transcriptome analysis was performed using the adrenal glands of male offspring (19-week-old) of naïve, saline or DEX exposed WKY dams. The RNA samples were analyzed using the Rat Gene 2.0 ST GeneChip microarray (ThermoFisher Scientific). This array covers over 28,000 protein coding transcripts from 23,500 Entrez genes, with a median of 22 probes per gene thereby providing excellent genome wide coverage. Whole transcriptome expression analysis of DEX exposed offspring compared with saline-treated controls revealed 190 significant DEGs (criteria: fold-change < − 1.5 and > 1.5; p-value < 0.05; false discovery rate < 0.1). 42 DEGs are currently unannotated or belong to the spliceosomal RNA family. The 142 annotated DEGs consisted of 109 upregulated genes and 35 downregulated genes (Fig. 1a), and illustrated as a volcano plot in Fig. 1b. The full list of upregulated and down-regulated DEGs based on fold change is presented in Supplementary Table 2. Importantly, the control comparison between the saline and naïve offspring resulted in no DEGs (Fig. 1a). Therefore, the naïve dataset was not considered for all further analyses.

### Principal component analysis (PCA) and unbiased exploratory grouping analysis (EGA)

PCA mapping was performed on the DEX (blue spheres) and saline (red cubes) transcriptome datasets (Fig. 1c). The three principal components accounted for 51.8% of the variance in the datasets. As expected this analysis revealed that the DEX and saline samples form discrete groupings, demonstrating that the DEX and saline groups have distinct global gene expression profiles. Next the microarray samples were analyzed using TCA’s EGA module, which enables analysis of relationships between transcriptome datasets without pre-defining known sample attributes and physiological parameters. Associating the sample IDs to the EGA plot demonstrates clear distribution of the samples into two distinct spatially disparate clusters: hypertensive DEX (blue spheres) and normotensive saline controls (red cubes) (Fig. 1d). The PCA plot reveals that whole transcriptomics datasets can be harnessed to predict blood pressure physiology based solely on gene expression profiles. Taken together, the PCA and EGA demonstrate that there are distinct underlying gene expression differences between prenatal DEX and saline exposed adrenal samples.

### Top dysregulated genes

The top 15 upregulated and downregulated genes in DEX versus saline dataset is presented in Fig. 1e,f respectively, ranked based on fold change. Some of the DEGs have been previously associated with the development of hypertension, including *Slc9A3*^31^ (solute carrier family 9 member A3; fold change = 4.55), *Hpgd*^32^ (hydroxyprostaglandin dehydrogenase; fold change = 2.56), *Pah*^7,30^ (phenylalanine hydroxylase; fold change = 2.10), *Fgf7*^33^ (fibroblast growth factor 7; fold change = 2.05) and *Cyp2e1*^34^ (cytochrome p450, family 2, subfamily e1; fold change = − 2.03) ^7,34,35^. However, the majority of top dysregulated genes are currently not implicated in the development of hypertension. In fact, there are numerous genes for orphan olfactory receptors and uncharacterized small nucleolar RNA molecules that are present in the highly downregulated genes (Fig. 1f and Supplementary Table 2). Taken together, the DEGs discovered in this study may potentially contribute to the identification of novel molecular mechanisms underlying the fetal programming of hypertension.

### RT-qPCR validation of transcriptome results

In order to confirm the transcriptomic results prior to further downstream bioinformatics analyses, selected genes from the microarray were cross-verified using RT-qPCR analysis. Genes analyzed included highly dysregulated as well as moderately expressed genes based on the transcriptome DEG list (Supplementary Table 2). Comparison of fold changes between the microarray and RT-qPCR data were equivalent (Fig. 1g), thereby providing confidence in the transcriptome data and its use in downstream bioinformatics analyses.

### Gene ontology and pathway enrichment analysis

**Figure 2:**
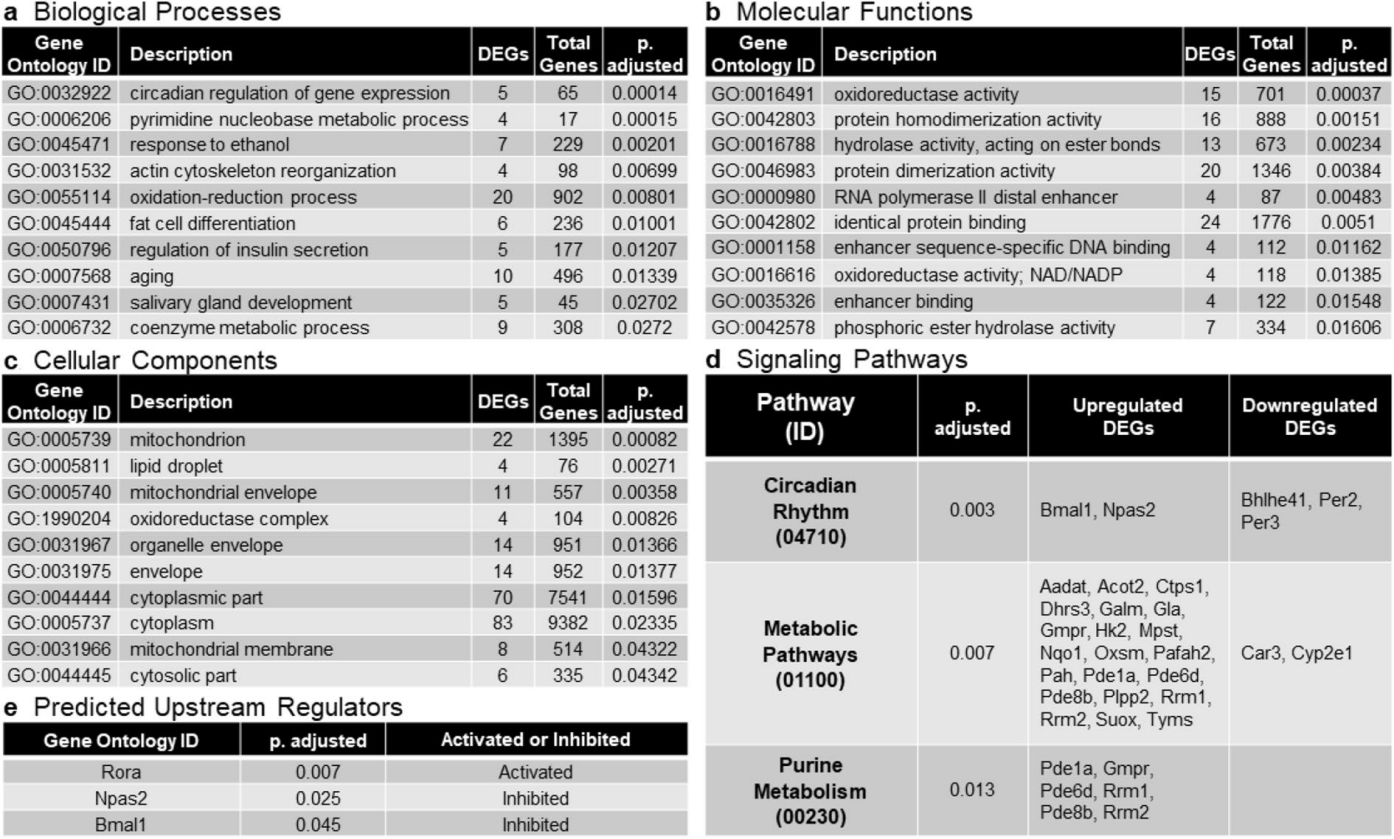
Summary of gene ontology (GO) and global pathway analyses in DEX adrenals relative to saline controls. Top 10 enriched GO terms categorized as (**a**) biological processes, (**b**) molecular functions and (**c**) cellular components are presented ranked by FDR p-value (*p*. adjusted). The number of DEGs identified in each GO term is provided along with the total number of genes annotated within the GO database. (**d**) Top signaling pathways (FDR p-value < 0.05) in DEX versus saline datasets identified by pathway enrichment analysis (iPathwayGuide). The upregulated and downregulated DEGs for each pathway is listed. (**e**) Predicted upstream regulators (FDR p-value < 0.05) identified by iPathwayGuide upstream regulator analysis. The activation or inhibition state is indicated. Activation or inhibition indicates that the upstream regulator is activated or inhibited respectively in DEX adrenals. Taken together, the GO and functional network analyses demonstrates that genes involved in circadian rhythm signaling are robustly dysregulated in DEX adrenals relative to saline controls.

In order to understand how the DEGs affect specific biological processes, GO enrichment analysis was performed. The iPG analysis package was used to hierarchically rank the DEGs within annotated GO units identified by the GO consortia database using iPG’s proprietary “impact analysis” method^28,29^. FDR correction was further applied to obtain GO terms with increased statistical significance. Top 10 enriched GO terms categorized as biological processes (Fig. 2a), molecular functions (Fig. 2b) and cellular components (Fig. 2c) are presented ranked by FDR p-value (*p*. adjusted). The number of DEGs identified in each GO term is provided along with the total number of genes annotated within the GO database. The top biological process was *circadian regulation of gene expression* followed by *pyrimidine nucleobase metabolic process*. Other noteworthy biological ontologies identified include *redox processes*, *fat cell differentiation*, and *regulation of insulin secretion*. The top molecular function was *oxidoreductase activity*, followed by a variety of pathways involved in metabolic processes. The top cellular components primarily involved the *mitochondria* and the *cytoplasm*. Here, the lack of nuclear components in the top list demonstrates that the majority of differences in the DEX versus saline dataset is due to dysregulation of genes which express proteins that contribute to cytoplasmic and mitochondrial functions.

The DEGs were also subjected to iPG’s pathway enrichment analysis to identify molecular signaling families that are dysregulated in DEX relative to saline samples. Global network enrichment analysis (Fig. 2d) demonstrated that genes involved in *circadian rhythm signaling* (p = 0.003) were most robustly dysregulated, followed by genes involved in *metabolic pathways* (p = 0.007) and *purine metabolism* (p = 0.013). The upregulated and downregulated DEGs for each pathway are also listed. *Circadian rhythm* genes *Bmal1* and *Npas2* were upregulated while the expression of *Bhlhe41*, *Per2*, and *Per3* were downregulated in DEX relative to saline. The majority of DEGs associated with *metabolic pathways* and *purine metabolism* were upregulated with limited downregulated genes (Fig. 2d). Taken together, the GO and pathway enrichment analysis demonstrates that prenatal DEX exposed adrenals have altered circadian rhythm signaling coupled with upregulation of the metabolic pathways.

### Upstream master regulator analysis

To identify master transcription regulators that can potentially explain the experimental DEGs, iPG’s predicted upstream regulators analysis was performed. Rora (p = 0.007), Npas2 (p = 0.025), and Bmal1 (p = 0.045) were identified as significant (FDR p-value < 0.05) upstream regulators (Fig. 2e). This analysis also classified the upstream regulators as activated (present) or inhibited (absent). For example, inhibited refers to master regulators that are predicted to be down-regulated in DEX samples relative to saline controls, and vice versa for activated regulators. Results suggest Rora is predicted to be activated while Npas2 and Bmal1 are inhibited (Fig. 2e). Interestingly, all three significantly predicted upstream master regulators are transcription factors that are involved in circadian rhythm signalling. Taken together, multiple DEG bioinformatics analyses demonstrate that genes involved in circadian rhythm signaling are robustly dysregulated in DEX adrenals relative to saline controls.

### Circadian rhythm signaling

**Figure 3:**
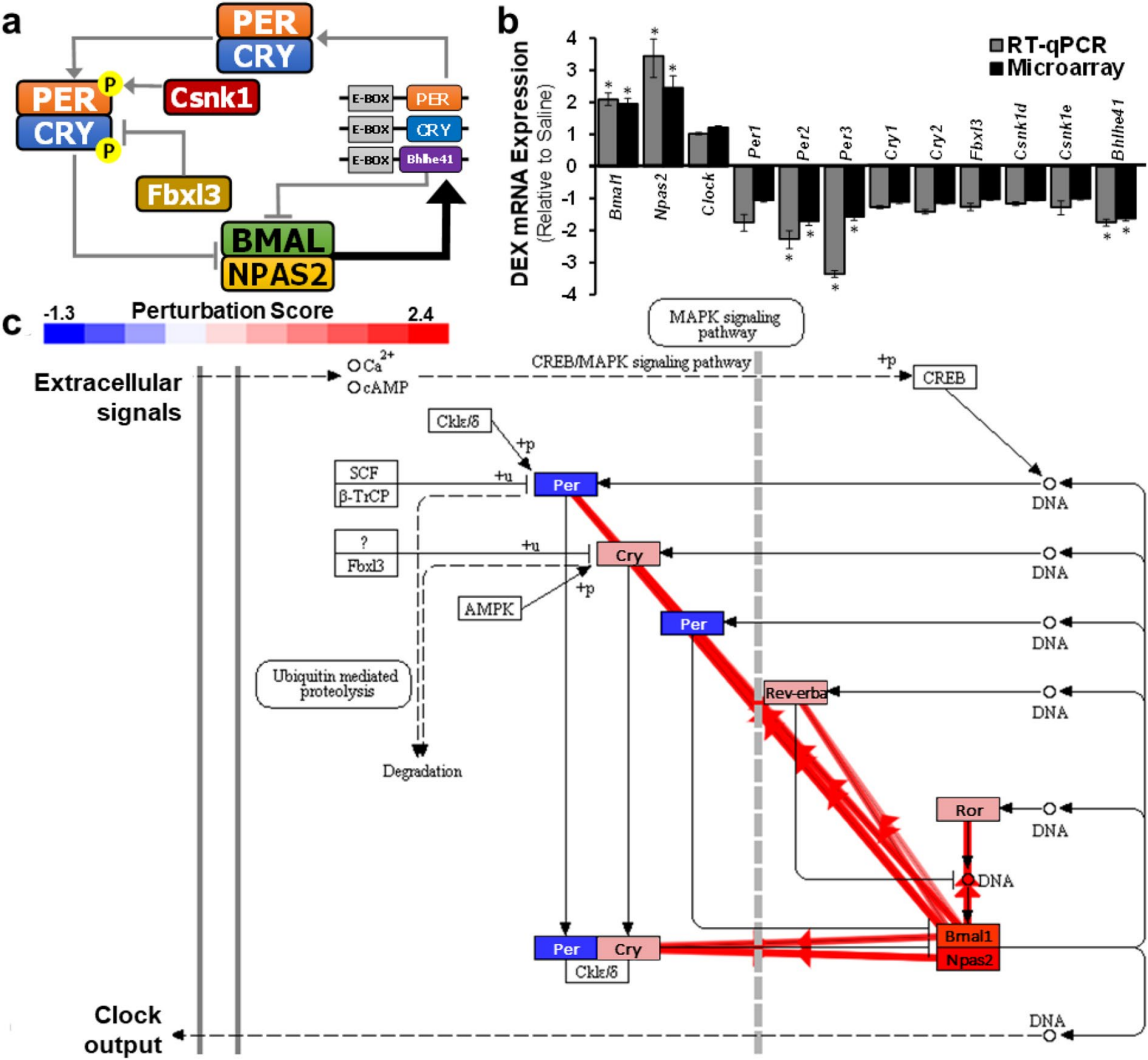
DEX adrenals demonstrate dysregulated expression of genes which control circadian rhythm signaling relative to saline controls. (**a**) Overview of the literature established circadian rhythm signaling. Bmal1 and Npas2/Clock forms a transcriptional activator complex which binds to E-Box promoter regions thereby driving the rhythmic expression of downstream genes such as *Per*, *Cry*, and *Bhlhe41*. Per and Cry forms a heterodimer and is phosphorylated by kinases (Csnk1). The phosphorylated Per-Cry complex drives the negative feedback loop by inhibiting further expression of *Bmal1* and *Npas2/Clock*. Similarly, Bhlhe41 re-enters nucleus and competitively inhibits the Bmal1-Npas2/Clock complex thereby supressing expression of *Per* and *Cry*. In addition, Fbxl3 promotes polyubiquitination of Cry proteins promoting their degradation. Expression of *Bmal1* and *Npas2/Clock* is highest during the day, while, *Per* and *Cry* expression peaks during the night. (**b**) Expression profiling of all known circadian rhythm signaling genes using RT-qPCR and microarray data. *Bmal1* and *Npas2* mRNA expression were significantly increased, while *Per2*, *Per3*, and *Bhlhe41* were downregulated in DEX relative to saline adrenals (n = 6; * p < 0.05). (**c**) The circadian rhythm pathway diagram obtained from iPathwayGuide (https://www.kegg.jp/kegg/kegg1.html) illustrating the computed perturbation from the DEG list of DEX adrenals relative to saline controls. The pathway diagram is overlayed with the computed perturbation of each gene. The perturbation accounts both for the measured fold change for each gene and the accumulated perturbation propagated from upstream regulators. The highest negative perturbation is shown in dark blue, while the highest positive perturbation is depicted in dark red. The legend describes the values on the gradient provided as a perturbation score. Coherent cascades are shown as red arrows. These cascades are sections of the pathway where the data describing the change in the gene expression is consistent with the established circadian signaling pathway from the literature.

The GO and pathway enrichment analysis, along with the upstream regulator analysis collectively establish that DEX adrenals demonstrate impaired expression of genes involved in circadian rhythm signaling. Figure 3a illustrates an overview of the main genes involved in circadian rhythm signaling. In order to fully characterize the circadian signalling pathway, gene expression for all known circadian rhythm signaling genes was analyzed by RT-qPCR (Fig. 3b). The RT-qPCR data corroborated the microarray data in showing that *Bmal1* and *Npas2* mRNA expression were significantly increased, while *Per2*, *Per3*, *Cry2* and *Bhlhe41were* downregulated in DEX relative to saline adrenals (n = 6; * p < 0.05). All other circadian rhythm genes tested (*Clock*, *Per1*, *Cry1*, *Fbxl3*, *Csnk1d* and *Csnk1e*) were not significantly different in the DEX and saline controls. In particular, Clock and Npas2 are paralogues, and both proteins can dimerize with Bmal1 to form a complex that drives the transcription of *Per*, *Cry*, and *Bhlhe41*^36^. The observation that a lack of change in gene expression in *Clock* suggests that *Bmal1*-*Npas2* complex is likely responsible for controlling circadian rhythm signaling in DEX exposed adrenals. In addition, the expression of *Bmal1* and *Npas2* should be reciprocal to *Per*, *Cry*, and *Bhlhe41*^37^. Indeed, *Per2*, *Per3*, *Cry2* and *Bhlhe41* are downregulated (Fig. 3b). Taken together, the circadian gene expression analysis in DEX exposed adrenals suggests underlying issues with the adrenal circadian rhythm.

Figure 3c illustrates the computed perturbation in the circadian rhythm pathway for the DEX adrenals relative to saline controls based on the transcriptome DEG list. The figure reports the computed perturbation score for each gene. A negative perturbation score (dark blue) indicates that the collective gene expression in the experimental dataset will cause a downregulation in the function of the gene, and vice versa for a positive score (dark red). The reported perturbation score is based on the combination of both the measured experimental fold change and the calculated accumulated perturbation from upstream genes. Here, the accumulated perturbation was calculated by taking into account the type, function, position, and interactions of each gene on the pathway by propagating downstream the measured expression change for each DEG^30^. Taken together, the perturbation score indicates that the circadian gene expression in DEX adrenals leads to the activation of Bmal1-Npas2 protein complex, which in turn inhibits the function of Per, while Cry, Ror, and Rev-erba are largely unaffected. Figure 3c also illustrates coherent cascades as red arrows. These cascades are sections of the pathway where the data describing the change in the gene expression is consistent with the established circadian signaling pathway from the literature. The abundance of red arrows initiating from the Bmal1-Npas2 protein complex illustrates that the genes involved in the circadian rhythm pathway in the DEX samples show consistent directionality as established by the GO databases.

### Gene expression differences in genetic versus DEX model of hypertension

**Figure 4:**
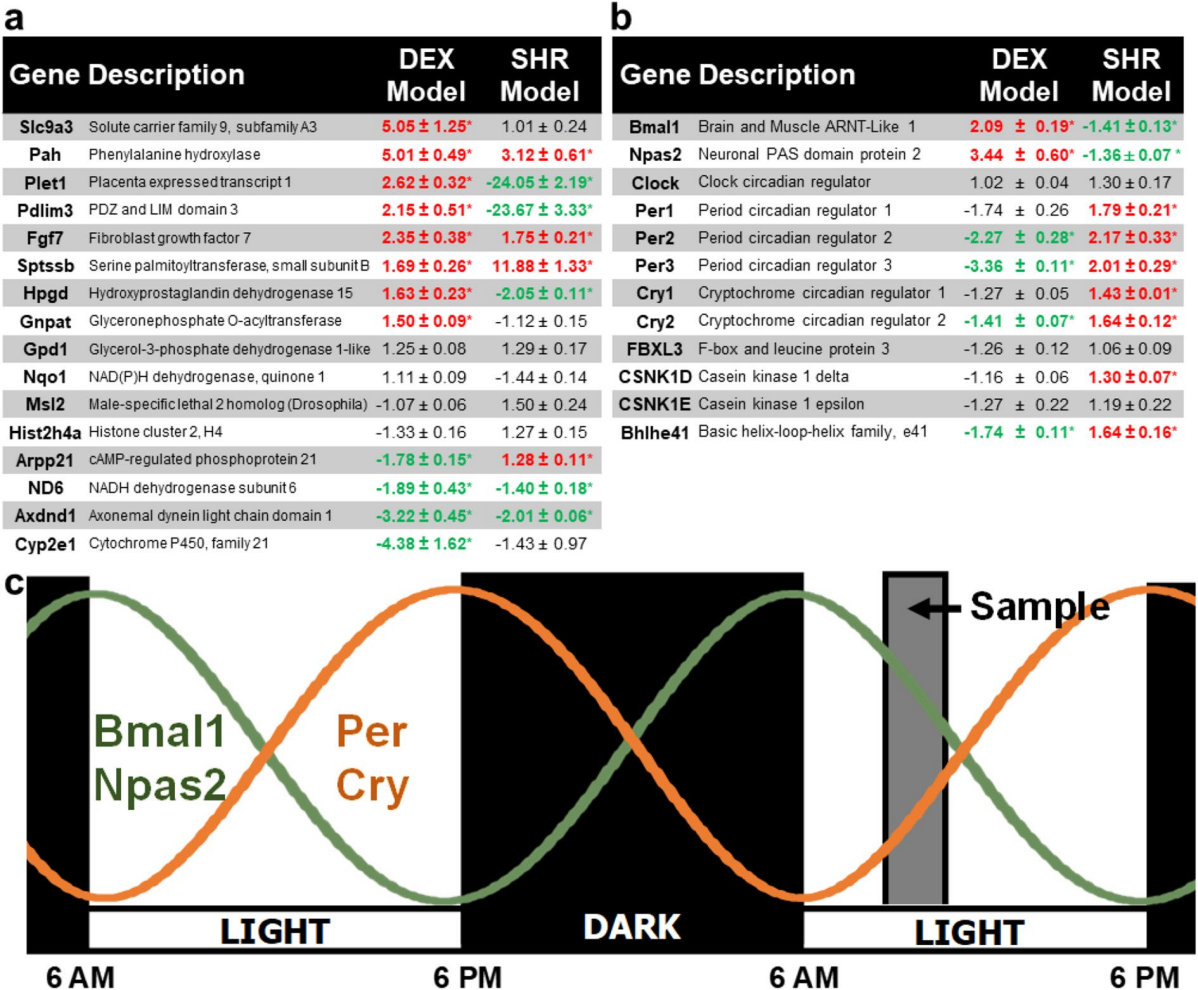
Comparison of 19-week-old male adrenal gene expression between the DEX model and the SHR model of hypertension using RT-qPCR. Values represent fold change ± standard error of means (n = 6; * p < 0.05; red = upregulated genes; green = downregulated genes). DEX fold change is relative to saline WKY controls, whereas SHR fold change is relative to naïve WKY controls. (**a**) Expression of 16 genes randomly selected from the DEX transcriptome microarray. This list includes highly dysregulated as well as moderate DEGs chosen at random. The fold change data shows that the DEX and SHR model demonstrate gene expression differences. (**b**) Expression profiling of all known circadian rhythm signaling genes in the DEX and SHR models of hypertension. (**c**) Literature established 24-h rhythmic gene expression of *Bmal1* (green line), *Npas2* (green line), *Per* (orange line) and *Cry* (orange line) for naïve WKY animals (*figure prepared by S. Tharmalingam*). The illustration depicts a 12-h light–dark cycle, with the light phase set between 6:00 am to 6:00 pm. The naïve WKY rats demonstrate peak *Bmal1* and *Npas2* expression during the dark/light transition, while *Per* and *Cry* expression peaks 12 h later during the light/dark transition. Adrenal samples were collected during 10 to 11 am (grey shaded region).

We were interested in determining how the gene expression profile in the prenatal DEX induced model of hypertension compares with the SHR genetic model of hypertension. RT-qPCR analysis of selected DEGs from the DEX model with the SHR model revealed numerous underlying gene expression differences (Fig. 4). The DEX fold change data is presented relative to saline WKY controls, and the SHR fold change data is shown relative to naïve WKY controls. As mentioned earlier, the transcriptome data for both the naïve and saline controls resulted in zero DEGs, showing that the gene expression is the same for both controls (Fig. 1a). 12 DEGs and 4 genes with similar expression from the DEX transcriptome study were randomly chosen for RT-qPCR analysis. The four genes that showed no difference in the DEX study (*Gpd1*, *Nqo1*, *Msl2*, and *Hist2h4a*) were also not changed in the SHR model. In contrast, only four of the twelve DEGs (*Pah*, *Fgf7*, *Nd6*, and *Axdnd1*) chosen from the DEX transcriptome showed similar fold change in both models (Fig. 4a) while the remaining 8 genes were significantly different (*Slc9a3*, *Plet1*, *Pdlim3*, *Sptssb*, *Hpgd*, *Gnpat*, *Arpp21*, and *Cyp2e1*). Interestingly, *Slc9a4* and *Cyp3e1* were the top upregulated and downregulated genes respectively in the DEX model, but the SHR model showed no difference in both gene expressions (Fig. 4a). More importantly, some genes had opposing expression patterns as in *Plet1* (fold change = 2.62 and − 24.05 for DEX and SHR models respectively) and *Pdlim3* (fold change = 2.15 and − 23.67 for DEX and SHR models respectively). Taken together, the DEX and SHR models demonstrate distinct gene expression profiles, revealing that stress mediated DEX model of developing hypertension differs significantly to the genetic SHR model in terms of adrenal gene expression.

Comparison of the circadian rhythm genes in both models also showed drastic differences (Fig. 4b). In contrast to the DEX model (Figs. 3b and 4b), the SHR model demonstrated decreased expression of *Bmal1* and *Npas2*, while *Per1*, *Per2*, *Per3*, *Cry1*, *Cry2*, *Bhlhe41* and *Csnk1D* were all upregulated compared to naïve WKY controls. Since both models demonstrate circadian system gene alterations compared to their respective controls, the overall data suggest that dysregulation of adrenal circadian rhythm may be an underlying mechanism for the development of hypertension.

## Discussion

This is the first study to report a global whole transcriptome analysis of GC programmed adrenal gland. Here we have identified several novel findings. First, we applied stringent transcriptomics parameters and identified 142 significant DEGs in DEX exposed adrenals compared to saline controls. This study is the first to associate these genes in GC mediated programming of the adrenal gland. Importantly, some of these DEGs may serve as putative biomarkers for the development of hypertension. Second, we utilized EGA to unbiasedly segregate samples into normotensive (saline controls) or hypertensive (DEX exposed group) based solely on the global whole transcriptome dataset without predefining the physiological parameters. We propose that this approach can be harnessed to predict an individual’s blood pressure physiology based solely on their gene expression profiles. Third and importantly, this study established that DEX adrenals have impaired circadian rhythm signaling based on multiple DEG bioinformatics platforms including GO enrichment, network pathway analysis, and upstream regulator prediction. Finally, we show that the adrenal glands of SHR rats demonstrated distinct gene expression profiles compared to the DEX programmed adrenals. Analysis of the circadian rhythm genes showed that the SHR animals also demonstrated circadian gene dysregulations compared to naïve WKY animals.

The circadian system consists of two parts: a central clock located in the suprachiasmatic nuclei (SCN) and peripheral clocks that are present in all organ systems^38,39^. The central clock obtains light–dark cues (zeitgeber) from the retina and relays this information to the peripheral clocks using humoral and neuronal signals to achieve circadian entrainment^40^. At the molecular level, the circadian system consists of transcription-translation feedback loops that drive rhythmic expression of core clock genes and their protein products (Fig. 3a). The literature established 24-h rhythmic gene expression pattern of *Bmal1*, *Npas2*, *Per* and *Cry* for naïve WKY animals is presented in Fig. 4c^36,37,39,41^. The illustration depicts a 12-h light–dark cycle, with the light phase set between 6:00 am to 6:00 pm as in the case with our experimental animals. Overview of the literature indicates that the naïve/saline WKY rats demonstrate peak *Bmal1* and *Npas2/Clock* expression (Fig. 4c; green line) during the dark/light transition, while *Per* and *Cry* demonstrates antiphasic expression (Fig. 4c; orange line) with peak levels 12 h later during the light/dark transition^36,37^. In this experiment, all adrenal samples were collected between 10 to 11 am. Comparison of circadian gene expression of the DEX model in the context of the 24-h rhythmic cycle shows that increased *Bmal1* and *Npas2*, and decreased *Per* and *Cry* is expected several hours earlier during the 6 am dark/light transition^37,41–46^. The opposite is true for the SHR model. Here, increased *Bmal1* and *Npas2*, and decreased *Per* and *Cry* relative to the naïve WKY animals is expected many hours later, closer to the 6 pm light/dark transition. Indeed, previous studies show that the SHR adrenals demonstrate circadian *phase advance*^39,41^, which corroborates the SHR circadian rhythm gene expression results from this study (Fig. 4b,c). Taken together, altered circadian rhythm entrainment may be an underlying molecular mechanism responsible for the development of the hypertensive phenotype observed in both the DEX and SHR models.

The Bmal1-Npas2/Clock transcriptional activator complex promotes numerous downstream effects including control of blood pressure regulation^46^. Indeed, various clock gene knockout models demonstrated blood pressure dysregulation. For example, *Bmal1* knockout animals exhibited reduced blood pressure and lacked circadian variation throughout a 24 h cycle^47^. Likewise, the *Clock* mutant mouse model showed dampened blood pressure and heart rate rhythm^48^. Similarly, *Per1* knockout^44^ lowered blood pressure while *Per2* mutants^42^ showed decreased diastolic blood pressure coupled with elevated heart rate. Taken together, these mutant studies demonstrate that circadian genes play an integral role in the control of blood pressure. Therefore, the dysregulation of circadian genes in the DEX exposed adrenal glands reported in this study may be an underlying programming mechanism driving the development of hypertension.

At the physiological level, it has been well established that blood pressure and plasma epinephrine levels exhibit circadian rhythm oscillations^40^. Blood pressure and epinephrine is lowest during night and undergoes a steep increase in the morning, peaking in the late afternoon. Similarly, circulating GC levels also demonstrate rhythmic levels. GCs have a complex ultradian rhythm composed of frequent episodes of GC secretion, with peak levels in the morning. This peak GC secretion is important for coordinating the central and peripheral clocks. However, human clinical studies have shown that children exposed to antenatal GC treatment lacked a cortisol awakening response and had a flatter diurnal slope^49^. This correlates with studies which report that offspring exposed to maternal undernutrition during fetal development showed a loss of diurnal variation in heart rate and blood pressure^50^. This is clinically significant since individuals that do not display a diurnal blood pressure response have been associated with hypertension and various adverse cardiovascular outcomes^46^. Taken together, these studies demonstrate that desynchronization of peripheral and central clocks promote the development of hypertension.

Studies using both circadian gene expression profiling and rhythmic behavioural locomotor activity in SHR animals demonstrated that the central SCN clock was *phase advanced* but the output rhythm was dampened compared to WKY controls. Analysis of the peripheral clock showed tissue-specific responses. The adrenal gland, colon, and plasma exhibited circadian *phase advance* while the liver was unaffected compared to control rats. The circadian *phase advance* in the SHR adrenal glands corresponded with the advanced rhythmic levels of serum corticosterone and aldosterone^41^, and dampened circadian blood pressure amplitudes^51^. At the behavioral level, the circadian dysregulation corresponds to previously established aberrant sleep/wake cycles in the SHR animals^52^. Interestingly, sleep disturbances contributes to a variety of diseases associated with fetal programming including insulin resistance, metabolic disorders and hypertension^53^. Therefore, given the desynchrony of circadian oscillators and its effect on the physiology of the SHR model, a thorough analysis of central and peripheral circadian systems along with sleep–wake regulation in the GC fetal programming model will be valuable. Furthermore, the circadian gene expression changes underlying the DEX and SHR models of hypertension identified in thus study may be applicable to human shift workers and jet lagged individuals that present with increased blood pressure^54,55^.

Apart from the circadian genes, several DEGs identified in the DEX programmed adrenals have been previously associated with the development of hypertension. For example, *Slc9A3* is the most highly upregulated gene in DEX adrenals relative to saline controls. This gene codes for a sodium-hydrogen (Na/H) transporter and its increased expression has been implicated in essential hypertension^4,31^. *HPGD* is another top upregulated gene and functions to inactivate prostaglandins. Prostaglandins A and E are potent vasodilators capable of lowering arterial pressure therefore increased inactivation of these molecules promote hypertension^32^. Likewise, *Pah* codes for the enzyme responsible for producing tyrosine, the precursor for the production of adrenal catecholamines which directly contributes to hypertension^4,7,35^. Furthermore, *Pah* has been recently identified as an adrenal stress sensitive gene and its expression is upregulated in adrenal glands of male rats exposed to chronic stress^56^. *Fgf7* is another robustly upregulated DEG which has been linked to hypertension^33^. Repressing *Fgf7* expression with mir-455-3p-1 inhibits pulmonary arterial hypertension by limiting RAS/ERK intracellular signaling. Finally, *Cyp2e1* and *Cxcl13* were the only downregulated genes previously associated with CVD^4^. *Cyp2e1* is a monooxygenase and reduced expression increases oxidative stress leading to the development of cardiac right ventricular failure^34^. In addition, numerous loss-of-function gene promoter polymorphisms in *Cyp2e1* have been identified in humans, and these mutations have been clinically associated with essential hypertension in men^57^. Other studies report that decreased *Cyp2e1* expression is associated with obesity^58^. *Cxcl13* is a chemokine which belongs to the inflammatory system and polymorphisms in its genotype has been associated with essential arterial hypertension^59^. Apart from the examples provided above, the majority of dysregulated genes presented in this study are currently not implicated in the development of hypertension. In fact, there are numerous genes for orphan olfactory receptors and uncharacterized small nucleolar/spliceosomal RNA molecules (Supplementary Table 2). Taken together, an in-depth analysis of the DEGs identified in thus study will be important for elucidating the putative hypertensive gene markers driving the fetal programming of hypertension.

Comparing select DEGs from the DEX programmed adrenals with the SHR adrenals demonstrated numerous underlying gene expression differences in both models (Fig. 4a). More importantly, we were interested in identifying dysregulated gene expression patterns that were similar in both models since hypertension is an underlying phenotype in both systems. We rationalized that this comparison will enable the identification of genes that are fundamental for the development of hypertension. The following genes showed similar expression patterns in both models: *Pah*, *Fgf7*, *Sptssb*, *Nd6*, *Axdnd1*. Here, *Pah*^4,34^ and *Fgf7*^33^ have been previously implicated in hypertension, therefore further analysis of *Sptssb*, *Nd6*, and *Axdnd1* may help elucidate whether these genes contribute to development of hypertension. In fact, a full whole transcriptome profiling of the SHR adrenals will greatly contribute to this type of analysis.

Pathway enrichment analysis showed that genes involved with *metabolic pathways* were significantly dysregulated in the DEX programmed adrenals. Analysis of the DEGs associated with this pathway revealed several subfamilies including *purine metabolism* (*Pde1a*, *Pde6d*, *Pde8b*, *Rrmr1*, *Rrm2*, *Gmpr*, *Ctps1*) and *mitochondrial metabolism* (*Gpd1l*, *Nqo1*, *Dhrs3*, *Suox*, *Hk2*, *Gla*, *Galm*, *Tomm40*, *Mrpl46*, and *Oxsm*). In addition, genes involved in *lipid regulation* and *steroid hormone production* were also dysregulated (*Plpp2*, *Sptssb*, *Cyp4f4*, *Medag*, *Acot2*, *Ldah*, Stard10). Interestingly, these pathways are predominantly driven by gene upregulation (Fig. 2d). Some studies report that cellular metabolism is under the control of circadian clocks^43,45,60^. Therefore perhaps the increased upregulation of these adrenal metabolic genes may be due to an underlying circadian rhythm dysregulation.

In conclusion, using unbiased whole-transcriptome analysis, we have identified several novel molecular gene expression biomarkers for GC mediated fetal programming. This study confirms that antenatal GC exposure reconfigures gene expression patterns at the cellular level thereby affecting multiple molecular pathways in adulthood. This permanent adaptation is likely the underlying mechanism which drives the development of various physiological disorders associated with fetal programming. Further studies utilizing global-scale approaches such as proteomics, metabolomics and epigenomics will be needed to fully characterize the molecular and physiological effects of fetal programming and its consequences on the development of adulthood diseases.

## Supplementary information

Supplementary Information
