# Supplementary material for: Whole transcriptome analysis of adrenal glands from prenatal glucocorticoid programmed hypertensive rodents
Source: Sci Rep. 2020 Oct 30;10:18755. doi: 10.1038/s41598-020-75652-y (PMC7603342; doi:10.1038/s41598-020-75652-y)
Supplement: Supplementary file 1 — Supplementary Information [file 41598_2020_75652_MOESM1_ESM.docx]

**Whole Transcriptome Analysis of Adrenal Glands from Prenatal Glucocorticoid Programmed Hypertensive Rodents**

Sujeenthar Tharmalingam^1,2,3,4,5^, Sandhya Khurana^1^, Alyssa Murray^1,2^, Jeremy Lamothe^1,4^, T.C. Tai^1,2,3,4^

*^1^Northern Ontario School of Medicine, Laurentian University, 935 Ramsey Lake Rd, Sudbury, ON, Canada, P3E 2C6*

*^2^Department of Biology, ^3^Department of Chemistry and Biochemistry, ^4^Biomolecular Sciences Program, Laurentian University, Sudbury, ON, Canada, P3E 2C6*

*^5^Health Sciences North Research Institute, Sudbury, ON, Canada, P3E 2H2*

***Corresponding Author:***

Dr. T.C. Tai

Northern Ontario School of Medicine

Laurentian University

935 Ramsey Lake Rd, Sudbury, ON, P3E 2C6.

Email: tc.tai@nosm.ca

Phone: (705) 662-7239

**Keywords:** fetal programming, prenatal, hypertension, adrenal, glucocorticoids, transcriptomics, gene expression profiling, microarray, circadian rhythm

**Running title:** prenatal glucocorticoid exposure alters circadian signaling in adulthood

**Supplementary Tables**

**Supplementary Table 1. Primer sequences and relevant information for genes analyzed via RT-qPCR.** T_A_ represents optimal annealing temperature.

| **Gene** | **Sequence (5'to 3’)** | **T_A_ (°C)** | **Accession ID** |
| --- | --- | --- | --- |
| Rpl32 | GGTGGCTGCCATCTGTTTTG | 60 | NM_013226.2 |
|  | GTTTCCGCCAGTTTCGCTTAAT |  |  |
| Rpl13 | AAGAAGGGAGACAGTTCTGCTG | 60 | NM_031101.1 |
|  | CTCCTCTTCCGTGATGGCTC |  |  |
| Gapdh | GTCATCCCAGAGCTGAACGG | 60 | NM_017008.4 |
|  | ATACTTGGCAGGTTTCTCCAGG |  |  |
| Hpgd | CAATAGCAGGGCTCATGCCT | 60 | NM_024390.2 |
|  | ACATTCAGTCTCACACCGCT |  |  |
| Slc9a3 | CCGCCTCAGCAACAAATCAG | 60 | NM_012654.1 |
|  | GAGCCTGTATCACATGTGTGTGG |  |  |
| Plet1 | ACAACCTACACAGTGACAGTCC | 60 | NM_001014209.1 |
|  | TGCCCACTGGTTTGTTGTCT |  |  |
| Pah | GCTGCTAAGCTAGACACCTCA | 60 | NM_012619.2 |
|  | CTTGTTTCCTGCCCAAAGTCT |  |  |
| Pdlim3 | GGTCATCACCAGGATCACTCC | 60 | NM_053650.1 |
|  | ACTCCGTACCAAAGCCATCG |  |  |
| Fgf7 | GGAGATGAGGAACAGCTACAAC | 60 | NM_022182.1 |
|  | CCTCATTGCATTCTTTCTTTGCAT |  |  |
| Gnpat | TTGCCCGTCCTTCCCTAGTA | 60 | NM_053410.1 |
|  | CTGAAAACACGTTGCGGAGG |  |  |
| Arntl | TGCCACTGACTACCAAGAAAGT | 60 | NM_024362.2 |
|  | ATTTTGTCCCGACGCCTCTT |  |  |
| Gpd1l | CTGTGTACAGCAGCTACCCG | 60 | NM_001191885.1 |
|  | AACAGCTGATCCCCAGTTCC |  |  |
| Nqo1 | CAGAAACGACATCACAGGGGA | 60 | NM_017000.3 |
|  | GGCCTTCCTTATACGCCAGA |  |  |
| Cyp2e1 | TTCACCAAGTTGGCAAAGCG | 60 | NM_031543.1 |
|  | CCTTGACAGCCTTGTAGCCA |  |  |
| Axdnd1 | TGGTGATCAGCGGATGATGG | 60 | XM_017599029.1 |
|  | TCAAGCCTCTGGTCAGTTCC |  |  |
| Hist2h4a | CTGTCACCTACACCGAGCAC | 60 | XM_578415.6 |
|  | AGGCCTAGACAAACGAGCAC |  |  |
| Arpp21 | GGTCACTCAGAGAGAAGAGATGG | 60 | NM_001135045.1 |
|  | CATGAGGGAGGCTGGGTAGA |  |  |
| Sptssb | TGCACCAAGCCTAGACCCT | 60 | NM_001271299.1 |
|  | CCATGACAGCACAGCAGGTAA |  |  |
| Npas2 | TCTTCTGAGAGGCAGCTTGAA | 60 | NM_001108214.2 |
|  | CAGGAGGGGCTAGGCACATT |  |  |
| Clock | AAGATGACACAGCGGAGGTC | 60 | NM_021856.2 |
|  | ACTGTGACATGCCTTGTGGG |  |  |
| Bmal1 | TGCCACTGACTACCAAGAAAGT | 60 | NM_024362.2 |
|  | ATTTTGTCCCGACGCCTCTT |  |  |
| Per1 | CTCTCCGCAACCAGGATACC | 60 | NM_001034125.1 |
|  | GCTAGGAGCTCTGAGAAGCG |  |  |
| Per2 | AAGTGACGGGTCGAGCAAAG | 60 | NM_031678.1 |
|  | CATGTCGGGCTCTGGAATGA |  |  |
| Per3 | CCACCCTCTCCAGGTCATGT | 60 | NM_023978.2 |
|  | CGCCACTGAAACCAAAACCAA |  |  |
| Cry1 | CCCACTAAAGCAAGGAAGAAGC | 60 | NM_198750.2 |
|  | CCCGCATGCTTTCGTATCAGTT |  |  |
| Cry2 | GGACTACATCCGGCGATACC | 60 | NM_133405.2 |
|  | GCCAATGATGCACTTAGCGG |  |  |
| Rev-ErbA | ATCTCGGTTGCCTCAGCATC | 60 | NM_001113422.1 |
|  | CTAGGCACCGAGCAGTAAGG |  |  |
| Mettl3 | ATGTGCAGCCCAACTGGATT | 60 | NM_001024794.1 |
|  | CTGTGCTTAAACCGGGCAAC |  |  |
| Fbxl3 | AGAGAAAGGAAGCTTTCCGCCC | 60 | NM_001100568.1 |
|  | TTCTCAGCAGTGCCTTCCTCA |  |  |
| Csnk1D | CACCTCACAGATTCCCGGTC | 60 | NM_139060.3 |
|  | AAGGAGAGTTCTCATCGGTGC |  |  |
| Csnk1E | CTCTGCAAAGGCTACCCCTC | 60 | NM_031617.1 |
|  | CTCTGCAAAGGCTACCCCTC |  |  |

**Supplementary Table 2.** Full list of DEGs in DEX exposed WKY adrenals relative to saline controls ranked by fold change. Genes with FDR p-value below 0.1, p-value below 0.05, and fold change <-1.5 or >1.5 were assigned as DEGs. Affymetrix probe IDs are indicated, along with NCBI gene symbols when available. There are 184 DEGs presented in this list. 42 genes are currently unannotated or belong to the spliceosomal RNA family and therefore do not have associated gene symbols.

| **ID** | **Gene Symbol** | **Description** | **Fold Change** | **p Value** | **FDR p Value** |
| --- | --- | --- | --- | --- | --- |
|  |  |  |  |  |  |
| 17627920 | Slc9a3 | solute carrier family 9, subfamily A, member 3 | 4.55 | 0.0004 | 0.0264 |
| 17844002 | Plet1 | placenta expressed transcript 1 | 3.01 | 0.0006 | 0.0313 |
| 17711526 | Hpgd | hydroxyprostaglandin dehydrogenase 15 (NAD) | 2.56 | 0.007 | 0.0898 |
| 17826665 | Plpp2 | phospholipid phosphatase 2 | 2.55 | 2.23E-06 | 0.0102 |
| 17791758 | Il23r | interleukin 23 receptor | 2.52 | 0.0008 | 0.0348 |
| 17674842 | N/A | U6 Spliceosomal RNA | 2.32 | 0.0026 | 0.0563 |
| 17747907 | Sptssb | serine palmitoyltransferase, small subunit B | 2.23 | 0.0048 | 0.077 |
| 17858614 | Npas2 | neuronal PAS domain protein 2 | 2.22 | 0.004 | 0.0709 |
| 17735148 | Ankrd34b | ankyrin repeat domain 34B | 2.22 | 0.0002 | 0.0225 |
| 17686241 | N/A | Uncharacterized non-coding transcript | 2.22 | 0.0001 | 0.0211 |
| 17827349 | Pah | phenylalanine hydroxylase | 2.10 | 0.0052 | 0.0792 |
| 17765771 | Fgf7 | fibroblast growth factor 7 | 2.05 | 0.0002 | 0.0226 |
| 17730976 | Tubb3 | tubulin, beta 3 class III | 2.02 | 0.0006 | 0.0317 |
| 17783907 | Vom1r82 | vomeronasal 1 receptor 82 | 2.01 | 0.0008 | 0.0356 |
| 17726248 | Lox | lysyl oxidase | 1.96 | 4.81E-05 | 0.017 |
| 17619710 | Arntl | aryl hydrocarbon receptor nuclear translocator-like | 1.95 | 0.0006 | 0.0332 |
| 17875518 | N/A | U6 Spliceosomal RNA | 1.94 | 0.0034 | 0.0662 |
| 17856217 | Gpd1l | glycerol-3-phosphate dehydrogenase 1-like | 1.9 | 9.90E-06 | 0.0111 |
| 17675579 | Medag | mesenteric estrogen-dependent adipogenesis | 1.89 | 0.0013 | 0.0425 |
| 17848299 | Als2cl | ALS2 C-terminal like | 1.85 | 9.49E-05 | 0.0185 |
| 17711471 | Aadat | aminoadipate aminotransferase | 1.85 | 0.0012 | 0.0411 |
| 17709864 | SNORD19B | Small nucleolar RNA SNORD19B | 1.83 | 0.0029 | 0.0605 |
| 17735984 | RGD1562550 | similar to hypothetical protein FLJ21657 | 1.83 | 0.0002 | 0.0225 |
| 17718710 | Gmpr | guanosine monophosphate reductase | 1.82 | 3.96E-05 | 0.0166 |
| 17843306 | Olr1245 | olfactory receptor 1245 | 1.81 | 0.001 | 0.0387 |
| 17619026 | Rrm1 | ribonucleotide reductase M1 | 1.79 | 0.0002 | 0.0229 |
| 17731134 | Gnpat | glyceronephosphate O-acyltransferase | 1.78 | 7.38E-06 | 0.0104 |
| 17799669 | Rraga | Ras-related GTP binding A | 1.78 | 6.97E-06 | 0.0104 |
| 17838231 | Ly6i | lymphocyte antigen 6 complex, locus I | 1.77 | 0.0038 | 0.0697 |
| 17764597 | Muc15 | mucin 15, cell surface associated | 1.76 | 0.0008 | 0.0352 |
| 17709760 | Pbrm1 | polybromo 1 | 1.76 | 0.0018 | 0.0484 |
| 17831385 | Mirlet7c-2 | microRNA let7c-2 | 1.76 | 0.0069 | 0.0895 |
| 17730286 | Hid1 | HID1 domain containing, transcript variant 2 | 1.76 | 6.63E-05 | 0.0179 |
| 17797920 | Cnr1 | cannabinoid receptor 1 (brain) | 1.75 | 0.0012 | 0.0415 |
| 17712775 | Myom2 | myomesin 2 | 1.75 | 0.0002 | 0.0216 |
| 17875534 | N/A | Non-coding transcript identified by NONCODE | 1.73 | 0.0001 | 0.0211 |
| 17813581 | N/A | Uncharacterized non-coding transcript | 1.73 | 0.0025 | 0.0556 |
| 17861793 | N/A | U6 Spliceosomal RNA | 1.72 | 0.0003 | 0.026 |
| 17854075 | Fam83b | family with sequence similarity 83, member B | 1.71 | 0.0001 | 0.0205 |
| 17826812 | Cyp4f4 | cytochrome P450, family 4, subfamily f, polypeptide 4 | 1.71 | 0.0047 | 0.0761 |
| 17698603 | Abhd4 | abhydrolase domain containing 4 | 1.71 | 0.0017 | 0.0464 |
| 17831256 | Parvb | parvin, beta | 1.70 | 5.48E-05 | 0.0176 |
| 17854347 | SNORA32 | Small nucleolar RNA SNORA32 | 1.70 | 0.0023 | 0.0541 |
| 17735439 | Mrps27 | mitochondrial ribosomal protein S27 | 1.70 | 3.28E-05 | 0.016 |
| 17733363 | Nqo1 | NAD(P)H dehydrogenase, quinone 1 | 1.69 | 1.23E-06 | 0.0102 |
| 17839177 | Kdelr3 | KDEL ERprotein retention receptor 3 | 1.67 | 0.0006 | 0.032 |
| 17804047 | Dhrs3 | dehydrogenase/reductase member 3 | 1.67 | 0.0023 | 0.0541 |
| 17881128 | Dctn3 | dynactin 3 | 1.67 | 4.31E-06 | 0.0104 |
| 17876638 | Tmem164 | transmembrane protein 164 | 1.66 | 0.0034 | 0.0655 |
| 17719993 | LOC686142 | similar to Ral guanine nucleotide dissociation | 1.66 | 0.0038 | 0.0688 |
| 17881621 | Sgk3 | serum/glucocorticoid regulated kinase family 3 | 1.66 | 0.0002 | 0.0232 |
| 17716283 | Fzd8 | frizzled class receptor 8 | 1.66 | 0.0015 | 0.0445 |
| 17707902 | Primpol | primase and DNA directed polymerase | 1.63 | 0.0006 | 0.0311 |
| 17833045 | Suox | sulfite oxidase | 1.62 | 0.0006 | 0.0329 |
| 17773850 | Pde1a | phosphodiesterase 1A, calmodulin-dependent | 1.62 | 2.12E-05 | 0.0143 |
| 17876898 | Mid1 | midline 1 | 1.61 | 0.0002 | 0.0225 |
| 17633661 | Mir344a-2 | microRNA 344a-2 | 1.61 | 0.0028 | 0.0589 |
| 17873566 | Rfk | riboflavin kinase | 1.61 | 0.0002 | 0.0225 |
| 17792516 | Hk2 | hexokinase 2 | 1.61 | 0.0001 | 0.0199 |
| 17878811 | Gla | galactosidase, alpha | 1.60 | 3.63E-06 | 0.0104 |
| 17837548 | RGD1564420 | similar to Hypothetical protein MGC31278 | 1.60 | 6.59E-05 | 0.0179 |
| 17701494 | Abhd6 | abhydrolase domain containing 6 | 1.59 | 0.0003 | 0.0244 |
| 17813587 | Galm | galactose mutarotase (aldose 1-epimerase) | 1.59 | 0.0007 | 0.0336 |
| 17830400 | Mpst | mercaptopyruvate sulfurtransferase | 1.59 | 7.45E-05 | 0.0181 |
| 17855772 | Dag1 | dystroglycan 1 glycoprotein 1 | 1.59 | 0.0014 | 0.0443 |
| 17723897 | Ccbe1 | collagen and calcium binding EGF domains 1 | 1.59 | 1.07E-05 | 0.0111 |
| 17815078 | Rrm2 | ribonucleotide reductase M2 | 1.58 | 0.008 | 0.0951 |
| 17715339 | Tubb2a | tubulin, beta 2A class IIa | 1.58 | 0.0003 | 0.026 |
| 17857303 | Rpl7l1 | ribosomal protein L7-like 1 | 1.57 | 0.0036 | 0.0671 |
| 17640498 | Tmem216 | transmembrane protein 216 | 1.57 | 0.0006 | 0.0325 |
| 17781204 | Steap2 | STEAP family member 2, metalloreductase | 1.57 | 0.0001 | 0.0199 |
| 17630338 | Tomm40 | translocase of outer mitochondrial membrane | 1.57 | 0.0022 | 0.0524 |
| 17743347 | Ddah1 | dimethylarginine dimethylaminohydrolase 1 | 1.57 | 0.0041 | 0.0718 |
| 17851432 | Ubash3b | ubiquitin associated and SH3 domain | 1.56 | 0.0002 | 0.0225 |
| 17873004 | Eda | ectodysplasin-A | 1.56 | 2.18E-06 | 0.0102 |
| 17879075 | Smarca1 | SWI/SNF related, matrix associated, actin | 1.56 | 0.001 | 0.039 |
| 17781681 | Met | MET proto-oncogene, receptor tyrosine kinase | 1.56 | 0.0006 | 0.0332 |
| 17635785 | Hpx | Hemopexin | 1.56 | 0.0022 | 0.0524 |
| 17764652 | Olr796 | olfactory receptor 796 | 1.56 | 0.0005 | 0.0293 |
| 17735269 | Wdr41 | WD repeat domain 41 | 1.56 | 0.0002 | 0.0218 |
| 17735400 | Enc1 | ectodermal-neural cortex 1 | 1.55 | 0.003 | 0.0616 |
| 17630418 | PVR | poliovirus receptor | 1.55 | 0.0009 | 0.0376 |
| 17842136 | SNORD5 | Small nucleolar RNA SNORD5 | 1.55 | 0.0031 | 0.0626 |
| 17711692 | Casp3 | caspase 3 | 1.54 | 0.0002 | 0.0226 |
| 17866187 | Pde6d | phosphodiesterase 6D, cGMP-specific, rod | 1.54 | 0.0002 | 0.0225 |
| 17802463 | Marcksl1 | MARCKS-like 1 | 1.54 | 0.0077 | 0.0941 |
| 17833710 | Reep6 | receptor accessory protein 6 | 1.54 | 0.0088 | 0.0995 |
| 17758543 | RGD1305587 | similar to RIKEN cDNA 2010107G23 | 1.53 | 0.0009 | 0.0376 |
| 17719593 | SNORA2 | Small nucleolar RNA SNORA2 | 1.53 | 0.0003 | 0.0252 |
| 17831400 | Ttc38 | tetratricopeptide repeat domain 38 | 1.53 | 0.0075 | 0.0927 |
| 17814639 | Ldah | lipid droplet associated hydrolase | 1.53 | 0.0045 | 0.0751 |
| 17853967 | Unc13c | unc-13 homolog C | 1.53 | 0.0022 | 0.0528 |
| 17631172 | Actn4 | actinin alpha 4 | 1.53 | 4.09E-05 | 0.0166 |
| 17634072 | Mrpl46 | mitochondrial ribosomal protein L46 | 1.53 | 0.0016 | 0.0463 |
| 17665910 | N/A | Uncharacterized non-coding transcript | 1.52 | 0.0007 | 0.0342 |
| 17802920 | Pafah2 | platelet-activating factor acetylhydrolase 2 | 1.52 | 0.0015 | 0.0444 |
| 17768185 | Aar2 | AAR2 splicing factor homolog | 1.52 | 0.0003 | 0.0251 |
| 17710889 | Lsm4 | LSM4 homolog, U6 small nuclear RNA | 1.52 | 6.14E-06 | 0.0104 |
| 17691208 | Nipsnap1 | nipsnap homolog 1 (C. elegans) | 1.52 | 0.0003 | 0.024 |
| 17867229 | Tyms | thymidylate synthetase | 1.52 | 0.0002 | 0.0225 |
| 17681515 | F5 | coagulation factor V (proaccelerin, labile factor) | 1.52 | 0.0006 | 0.0331 |
| 17820353 | Slc30a6 | solute carrier family 30 (zinc transporter) | 1.52 | 3.96E-05 | 0.0166 |
| 17875077 | Abcd1 | ATP-binding cassette, subfamily D (ALD) 1 | 1.52 | 3.86E-05 | 0.0166 |
| 17676504 | Rpl31l4 | ribosomal protein L31-like 4 | 1.52 | 0.0001 | 0.0193 |
| 17669145 | Lrrc58 | leucine rich repeat containing 58 | 1.51 | 0.0031 | 0.0624 |
| 17716218 | N/A | Uncharacterized non-coding transcript | 1.51 | 0.0088 | 0.0997 |
| 17835345 | Socs2 | suppressor of cytokine signaling 2 | 1.51 | 0.0002 | 0.0234 |
| 17874884 | Tmem185a | transmembrane protein 185A | 1.51 | 0.0003 | 0.0252 |
| 17788778 | Psmc2 | proteasome 26S subunit, ATPase 2 | 1.51 | 1.09E-05 | 0.0111 |
| 17618808 | Stard10 | StAR-related lipid transfer domain 10 | 1.51 | 2.91E-05 | 0.0158 |
| 17769116 | Cse1l | CSE1 chromosome segregation 1-like (yeast) | 1.51 | 0.0027 | 0.0578 |
| 17678672 | Triap1 | TP53 regulated inhibitor of apoptosis 1 | 1.51 | 4.76E-05 | 0.017 |
| 17744336 | Pde8b | phosphodiesterase 8B | 1.51 | 0.0006 | 0.032 |
| 17817302 | Acot2 | acyl-CoA thioesterase 2 | 1.51 | 0.0071 | 0.0906 |
| 17697472 | Oxsm | 3-oxoacyl-ACP synthase, mitochondrial | 1.50 | 4.30E-05 | 0.0166 |
| 17810138 | Ctps1 | CTP synthase 1 | 1.50 | 5.36E-06 | 0.0104 |
| 17790391 | Mir29a | microRNA 29a | 1.50 | 0.001 | 0.039 |
| 17698372 | N/A | Uncharacterized non-coding transcript | -1.50 | 0.006 | 0.0844 |
| 17794919 | Ptms | Parathymosin | -1.50 | 0.0028 | 0.0591 |
| 17698476 | LOC103693835 | uncharacterized LOC103693835 mRNA | -1.51 | 0.007 | 0.0898 |
| 17861310 | SCARNA6 | Small Cajal body specific RNA 6 | -1.52 | 0.0068 | 0.0885 |
| 17763746 | N/A | Uncharacterized non-coding transcript | -1.53 | 0.0075 | 0.093 |
| 17611549 | RNU2-19 | Small nucleolar RNA U2-19 | -1.53 | 5.55E-05 | 0.0176 |
| 17837818 | N/A | Uncharacterized non-coding transcript | -1.53 | 0.0017 | 0.0473 |
| 17723822 | N/A | Uncharacterized non-coding transcript | -1.53 | 0.0007 | 0.0332 |
| 17693699 | N/A | Non-coding transcript identified by NONCODE | -1.53 | 0.0025 | 0.0556 |
| 17666901 | Olr1566 | olfactory receptor 1566 | -1.54 | 0.0048 | 0.0765 |
| 17632721 | SNORD34 | Small nucleolar RNA SNORD34 | -1.54 | 0.0007 | 0.0343 |
| 17828197 | N/A | Non-coding transcript identified by NONCODE | -1.54 | 0.0044 | 0.0742 |
| 17652486 | N/A | Uncharacterized non-coding transcript | -1.54 | 0.0018 | 0.0474 |
| 17681327 | N/A | Non-coding transcript identified by NONCODE | -1.55 | 0.001 | 0.0387 |
| 17752111 | SNORD45 | Small nucleolar RNA SNORD45 | -1.55 | 0.0074 | 0.0923 |
| 17791708 | N/A | Uncharacterized non-coding transcript | -1.55 | 0.0037 | 0.068 |
| 17859651 | SNORD11B | Small nucleolar RNA SNORD11B | -1.56 | 0.0015 | 0.0452 |
| 17693246 | Cxcl13 | chemokine (C-X-C motif) ligand 13 | -1.56 | 0.001 | 0.0378 |
| 17761757 | Olr397 | olfactory receptor 397 | -1.56 | 0.0025 | 0.0559 |
| 17866453 | Per2 | period circadian clock 2 | -1.57 | 0.0055 | 0.0808 |
| 17731373 | RNA5S1 | 5S ribosomal RNA | -1.57 | 0.0002 | 0.0225 |
| 17624341 | Olr357 | olfactory receptor 357 | -1.57 | 0.0067 | 0.0879 |
| 17626953 | N/A | Uncharacterized non-coding transcript | -1.57 | 5.94E-05 | 0.0179 |
| 17817765 | N/A | U1 Spliceosomal RNA | -1.58 | 0.0031 | 0.0627 |
| 17883013 | N/A | Uncharacterized non-coding transcript | -1.58 | 0.0024 | 0.0545 |
| 17641711 | N/A | U6 Spliceosomal RNA | -1.59 | 0.0051 | 0.0784 |
| 17610505 | Epm2a | epilepsy, progressive myoclonus type 2A | -1.60 | 0.0009 | 0.0371 |
| 17832979 | Rpl41 | ribosomal protein L41 | -1.60 | 0.0006 | 0.032 |
| 17648368 | Olr1511 | olfactory receptor 1511 | -1.60 | 0.0049 | 0.0776 |
| 17790359 | N/A | Non-coding transcript identified by NONCODE | -1.61 | 0.0007 | 0.0334 |
| 17702426 | N/A | Uncharacterized non-coding transcript | -1.61 | 0.0008 | 0.0356 |
| 17856096 | Arpp21 | cAMP-regulated phosphoprotein 21 | -1.61 | 0.0015 | 0.0452 |
| 17670381 | SNORD66 | Small nucleolar RNA SNORD66 | -1.61 | 0.0006 | 0.0327 |
| 17839270 | SNORD83B | Small nucleolar RNA U83B | -1.64 | 0.0013 | 0.0423 |
| 17717017 | N/A | Uncharacterized non-coding transcript | -1.64 | 0.0007 | 0.0332 |
| 17611071 | SNORD100 | Small nucleolar RNA SNORD100 | -1.64 | 0.0003 | 0.024 |
| 17851230 | Olr1253 | olfactory receptor 1253 | -1.64 | 0.0002 | 0.0227 |
| 17796582 | Bhlhe41 | basic helix-loop-helix family, member e41 | -1.64 | 0.0004 | 0.0277 |
| 17654066 | Olr1372 | olfactory receptor 1372 | -1.65 | 0.0018 | 0.0477 |
| 17880845 | N/A | Uncharacterized non-coding transcript | -1.66 | 0.0012 | 0.0417 |
| 17854574 | SNORD50 | Small nucleolar RNA SNORD50 | -1.66 | 0.0008 | 0.0348 |
| 17812917 | Per3 | period circadian clock 3 | -1.66 | 0.0014 | 0.043 |
| 17656034 | SNORA42 | Small nucleolar RNA SNORA42/SNORA80 family | -1.67 | 0.0031 | 0.0623 |
| 17619216 | Olr200 | olfactory receptor 200 | -1.69 | 0.0054 | 0.0804 |
| 17690410 | N/A | Uncharacterized non-coding transcript | -1.69 | 0.0079 | 0.0951 |
| 17882841 | N/A | Uncharacterized non-coding transcript | -1.69 | 0.0079 | 0.0951 |
| 17848896 | SNORA62 | Small nucleolar RNA SNORA62 | -1.69 | 0.0029 | 0.0607 |
| 17717650 | N/A | Uncharacterized non-coding transcript | -1.71 | 0.0019 | 0.0497 |
| 17791387 | Hoxa6 | homeobox A6 | -1.75 | 0.0015 | 0.0444 |
| 17704920 | N/A | U1 Spliceosomal RNA | -1.78 | 0.0068 | 0.0891 |
| 17869590 | N/A | Uncharacterized non-coding transcript | -1.83 | 0.0007 | 0.0336 |
| 17773945 | N/A | Non-coding transcript identified by NONCODE | -1.85 | 0.0041 | 0.0723 |
| 17646717 | SNORD65 | Small nucleolar RNA SNORD65 | -1.94 | 0.0007 | 0.0332 |
| 17724639 | N/A | U1 Spliceosomal RNA | -1.98 | 0.0023 | 0.0534 |
| 17762517 | N/A | U2 Spliceosomal RNA | -2.00 | 0.0009 | 0.0371 |
| 17652193 | N/A | U11 Spliceosomal RNA | -2.00 | 0.0004 | 0.0277 |
| 17876552 | N/A | U11 Spliceosomal RNA | -2.03 | 0.0004 | 0.0265 |
| 17622015 | Cyp2e1 | cytochrome P450, family 2, subfamily e, 1 | -2.03 | 0.0008 | 0.0359 |
| 17862492 | N/A | U1 Spliceosomal RNA | -2.06 | 0.0057 | 0.0821 |
| 17799063 | N/A | U6 Spliceosomal RNA | -2.10 | 0.0074 | 0.0923 |
| 17792483 | N/A | U11 Spliceosomal RNA | -2.18 | 0.0002 | 0.0224 |
| 17858089 | N/A | Uncharacterized non-coding transcript | -2.18 | 0.0002 | 0.0224 |
| 17695867 | N/A | U2 Spliceosomal RNA | -2.18 | 0.0062 | 0.0851 |
| 17878689 | N/A | U1 Spliceosomal RNA | -2.26 | 0.0014 | 0.0433 |
| 17722610 | N/A | Non-coding transcript identified by NONCODE | -2.29 | 0.0015 | 0.0449 |
| 17685599 | Axdnd1 | axonemal dynein light chain domain 1 | -2.39 | 0.0018 | 0.0474 |
| 17760345 | SNORD24 | Small nucleolar RNA SNORD24 | -2.99 | 0.0037 | 0.0682 |
